# Mutational Analysis of Ocriplasmin to Reduce Proteolytic and Autolytic Activity in *Pichia pastoris*

**DOI:** 10.1186/s12575-020-00138-0

**Published:** 2020-12-13

**Authors:** Roghayyeh Baghban, Safar Farajnia, Younes Ghasemi, Reyhaneh Hoseinpoor, Azam Safary, Mojtaba Mortazavi, Nosratollah Zarghami

**Affiliations:** 1 Medical Biotechnology Department, Faculty of Advanced Medical Science, Tabriz University of Medical Sciences, Tabriz, Iran; 2 Research Committee, Tabriz University of Medical Sciences, Tabriz, Iran; 3 Poostchi Ophthalmology Research Center, Shiraz University of Medical Sciences, Shiraz, Iran; 4 Biotechnology Research Center, Tabriz University of Medical Sciences, Daneshgah Ave, Tabriz, Iran; 5 Drug Applied Research Center, Tabriz University of Medical Sciences, Tabriz, Iran; 6 Department of Pharmaceutical Biotechnology, Faculty of Pharmacy and Pharmaceutical Sciences Research Center, Shiraz University of Medical Science, Shiraz, Iran; 7 Department of Biotechnology, School of Advanced Technologies in Medicine, Shahid Beheshti University of Medical Sciences, Tehran, Iran; 8 Connective Tissue Diseases Research Center, Tabriz University of Medical Sciences, Tabriz, Iran; 9 Department of Biotechnology, Institute of Science and High Technology and Environmental Science, Graduate University of Advanced Technology, Kerman, Iran

**Keywords:** Ocriplasmin, *Pichia pastoris*, Site-directed mutagenesis, Proteolytic activity, Autolytic activity

## Abstract

**Background:**

Ocriplasmin (Jetrea) is using for the treatment of symptomatic vitreomacular adhesion. This enzyme undergoes rapid inactivation and limited activity duration as a result of its autolytic nature after injection within the eye. Moreover, the proteolytic activity can cause photoreceptor damage, which may result in visual impairment in more serious cases.

**Results:**

The present research aimed to reduce the disadvantages of ocriplasmin using site-directed mutagenesis. To reduce the autolytic activity of ocriplasmin in the first variant, lysine 156 changed to glutamic acid and, in the second variant for the proteolytic activity reduction, alanine 59 mutated to threonine. The third variant contained both mutations. Expression of wild type and three mutant variants of ocriplasmin constructs were done in the *Pichia pastoris* expression system. The mutant variants were analyzed in silico and in vitro and compared to the wild type. The kinetic parameters of ocriplasmin variants showed both variants with K156E substitution were more resistant to autolytic degradation than wild-type. These variants also exhibited reduced K_cat_ and V_max_ values. An increase in their Km values, leading to a decreased catalytic efficiency (the K_cat_/K_m_ ratio) of autolytic and mixed variants. Moreover, in the variant with A59T mutation, K_cat_ and V_max_ values have reduced compared to wild type. The mix variants showed the most increase in Km value (almost 2-fold) as well as reduced enzymatic affinity to the substrate. Thus, the results indicated that combined mutations at the ocriplasmin sequence were more effective compared with single mutations.

**Conclusions:**

The results indicated such variants represent valuable tools for the investigation of therapeutic strategies aiming at the non-surgical resolution of vitreomacular adhesion.

## Background

Symptomatic vitreomacular adhesion or vitreomacular traction may cause edema, macular distortion and, form macular holes. This sight-threatening condition relates to decreased visual acuity, photopsia and, metamorphopsia [1–4]. Vitreomacular traction spontaneously clears up in 10–35% of the cases [5, 6]; otherwise, it can progress into visual impairment [1].

Vigilant monitoring and pars plana vitrectomy constitute standard procedures for managing symptomatic vitreomacular adhesion [1, 3]. Given the risk of damage to the retina caused by the surgery [7], proteolytic enzymes such as plasmin have been examined for the enzymatic release of retinal traction [8].

Vitreomacular adhesion can be treated and full-thickness macular holes are closed as a result of the activities of the recombinant protease ocriplasmin against the components of the extracellular matrix that form part of the vitreoretinal interface [3, 9–11]. Two pivotal Phase III trials, including ocriplasmin for Intravitreous Injection-Traction Release without Surgical Treatment [MIVI-TRUST], confirmed the efficacy and safety of ocriplasmin in treating symptomatic vitreomacular adhesion. Therefore, the Food and Drug Administration approved ocriplasmin in 2012 for treating vitreomacular traction [4, 9].

Ocriplasmin, as a serine protease, is a truncated form of plasmin that has maintained its enzymatic properties [10, 12]. It is a powerful collagenase activator with proteolytic activity against a variety of components of the vitreoretinal interface, including laminin and fibronectin [13]. Plasmin, the enzyme from which ocriplasmin is derived, is a trypsin family member and cuts proteins at the basic amino acids, lysine and arginine [14]. Ocriplasmin has some advantages over plasmin; most especially, ocriplasmin is significantly more stable than plasmin. It is also smaller than plasmin, which leads to enhanced penetration into the tissues [13].

Active ocriplasmin comprises 2 polypeptides of 230 and 19 residues, which are connected by 2 disulfide bonds. Four intra-chain disulfide bonds stabilize the domain of 230-residue [8]. Despite obviating the risks associated with the surgery, pharmacologic vitreolysis with ocriplasmin cannot be considered free of any risks [15].

The autolytic and proteolytic nature of this enzyme at physiologic pH limits its duration of activities. Proteolytic activity can cause photoreceptor damage, which may result in visual impairment in more serious cases [16–18]. Moreover, the autolytic degradation of ocriplasmin causes its fast inactivation after vitreous injection [19, 20].

As a highly-promising biocatalyst engineering method, protein engineering can be used to promote enzyme stability and efficiency [21]. Rapid advancements in biological sciences have led up to state-of-the-art protein engineering methods, especially recombinant DNA technology. The random types of these approaches include random mutagenesis and evolutionary techniques such as DNA shuffling. The rational methods include site-directed mutagenesis [22, 23] as an effective yet simple method that introduces special amino acids into target genes using 2 oligonucleotide primers with the desired mutations that complement the opposite strands of a double-stranded DNA template [24–26].

The autolytic cleavage of ocriplasmin is limited to 3 positions, i.e. R177-V178, K166-V167 and, K156-E157 as the most important autolytic cleavage site that greatly increases the sensitivity of the other two sites [27]. Besides, the functional activity of plasminogen was found to decrease in dysplasminogenemia. Mutation of Ala601Thr was also described in dysplasminogenemia. The substitution of alanine 601 for threonine was found to play a key role in reducing the activity (alanine 601 in plasminogen is equivalent to alanine 59 in ocriplasmin) [28, 29].

In recent years, *Pichia pastoris* (renamed as *Komagataella phafii*) [30] expression system has been considered to be an outstanding host for generating proteins from different sources. The eukaryotic *P. pastoris* has numerous advantages over prokaryotic expression systems such as ease of genetic manipulation, high expression levels, high-density culture, easier upscaling, and powerful methanol-regulated alcohol oxidase promoter (AOX1). Also, proteins expressed by *Pichia* are commonly soluble and properly folded [31, 32].

This study designed and generated three mutant variants of ocriplasmin for reducing autolytic and proteolytic activities after in silico analysis. In the autolytic variant, lysine 156 was mutated to glutamic acid; in the proteolytic variant alanine 59 changed to threonine, and the autolytic-proteolytic variant (mixed variant) contained both of above-mentioned mutations. All the variants were cloned and expressed in the *Pichia pastoris* expression system. The autolytic/proteolytic activity of variants was measured following activation with urokinase. The kinetic parameters of the wild-type and mutant variants of ocriplasmin with the substrate S-2403 were calculated based on the Michaelis–Menten plot. Significant resistance to autolytic inactivation and proteolytic activity was reported in ocriplasmin variants using different assessment methods.

## Results

### In Silico Analysis of Ocriplasmin Variants

Structural analyses performed using the PIC web server revealed that the substitution of alanine 59 for threonine (A59T) significantly reduces proteolytic activities and the substitution of lysine156 for glutamic acid (K156E) notably affects the autolytic function of the enzyme.

**Fig. 1:**
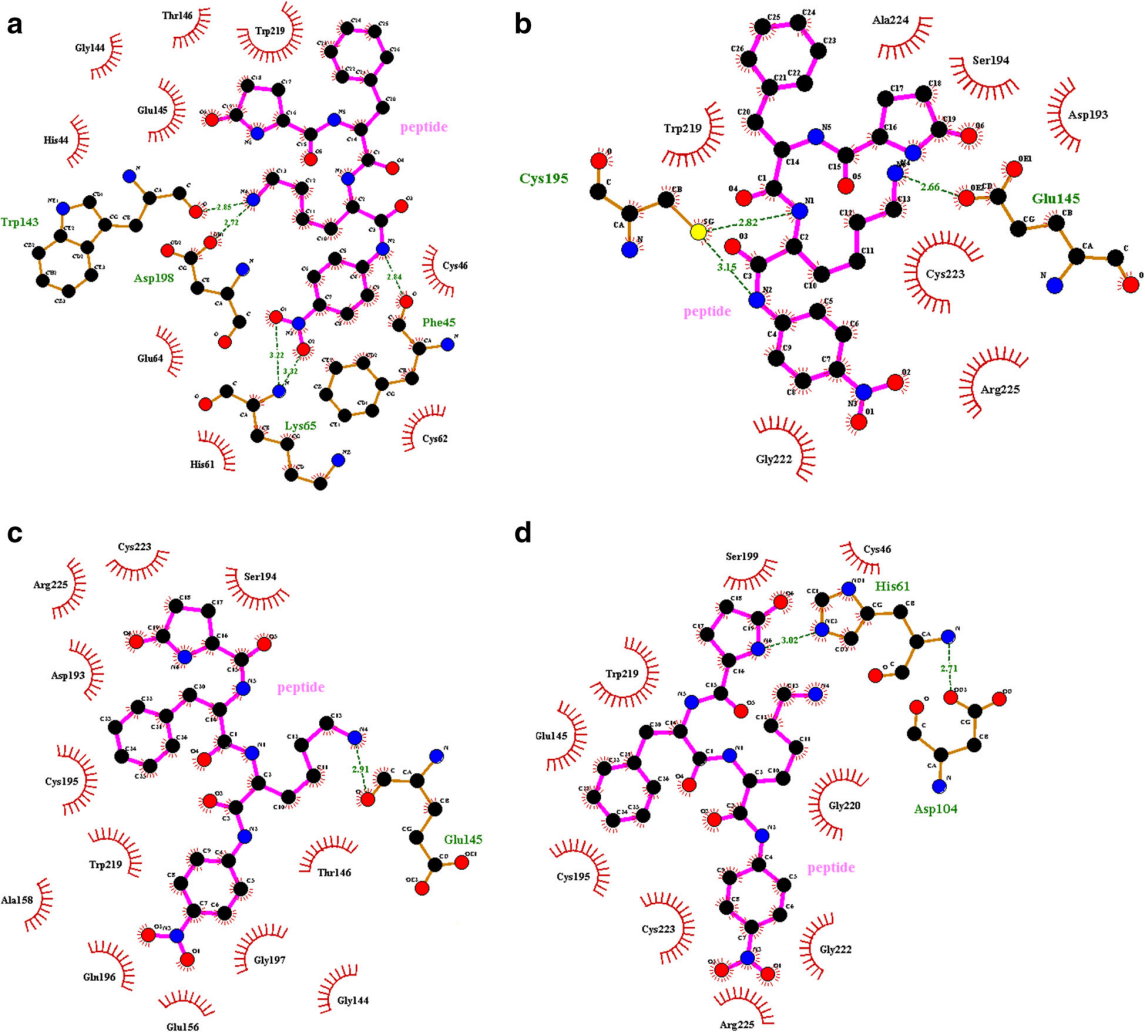
Molecular interactions between S-2403 substrate and amino acids of ocriplasmin presented by 2-D mode. The formed hydrogen bonds among amino acids and substrate presented by green spheres in, **a** peptide-wild type ocriplasmin, **b** peptide-proteolytic type ocriplasmin, **c** peptide-autolytic type ocriplasmin and, **d** peptide- mixed type ocriplasmin in the docked systems

The docking results showed the lower binding affinity of the mutant variants than that of wild-type ocriplasmin. The mutant variants were found to be able to bind substrates with high to low affinity. The molecular docking simulations of the substrate S-2403 with different variants of ocriplasmin showed that the minimum free binding energy (ΔG) between them in the high number conformations cluster were respectively − 7.67, − 6.93, − 6.75, − 5.84 kcal/mol for substrate-wild type ocriplasmin, substrate-autolytic type ocriplasmin, substrate-proteolytic type ocriplasmin, and substrate-mixed type ocriplasmin. Figure 1a-d shows two-dimensional amino acids that interacted and made hydrogen bonds with ocriplasmin variants. The images were prepared using Lig Plot v1.4.4 software.

Computational simulations using RMSD, RMSF, and center of mass average distances showed more affinity of the substrate S-2403 to the wild-type than mutant variants. The average RMSD was respectively obtained as 0.33, 0.34, and 0.39 nm for the three mutant variants of ocriplasmin in the complex with the substrate-proteolytic, substrate-autolytic, and substrate-mixed type, and an average RMSD of 0.28 nm was derived for the substrate-wild type ocriplasmin throughout the simulation.

**Fig. 2:**
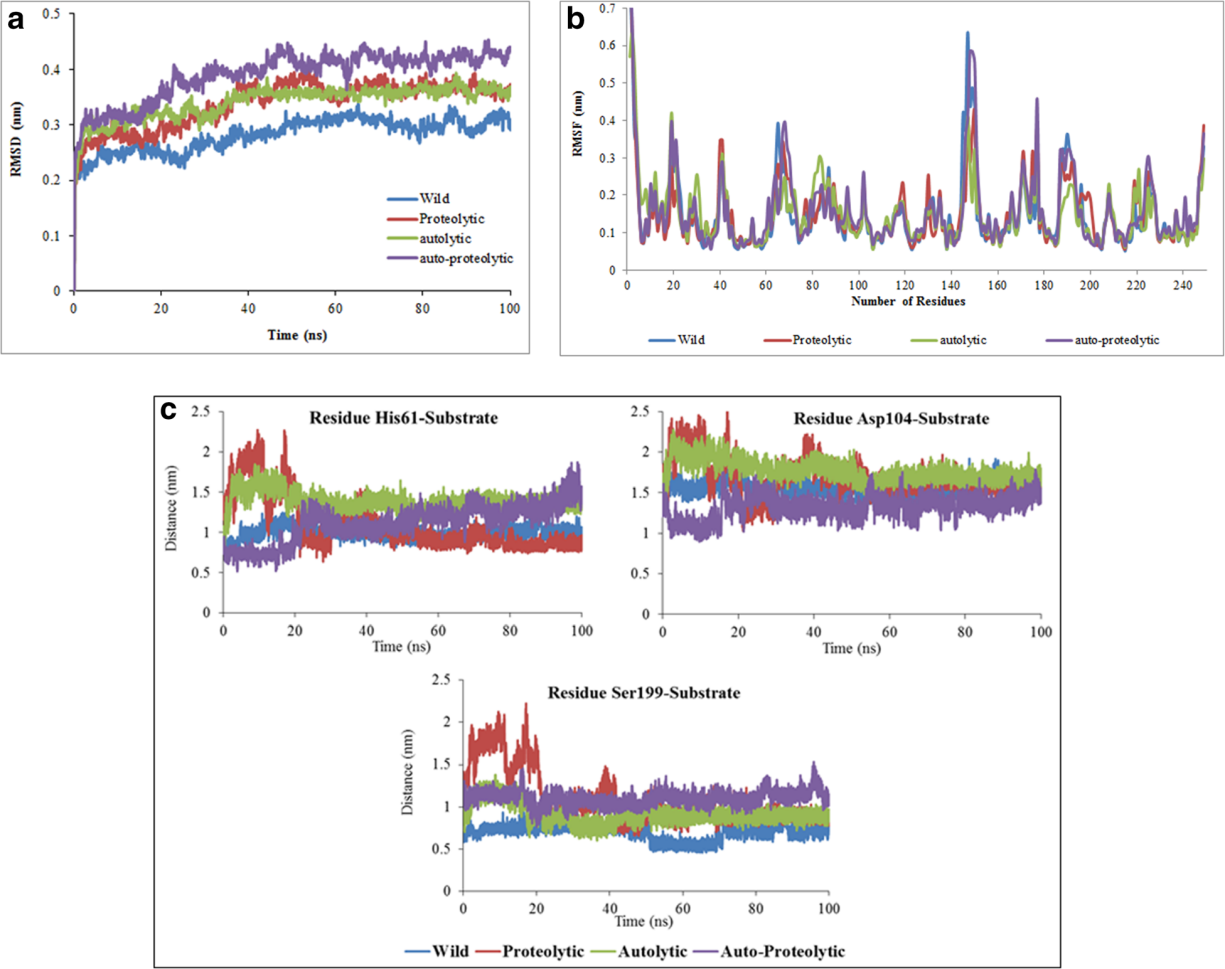
The analysis of RMSD (root mean square deviation), RMSF (root-mean-square fluctuations), and center of mass distance between ocriplasmin and substrate throughout the molecular dynamic simulation. **a** Backbone RMSD are shown as a function of time for wild type and mutant variants. **b** RMSF of the backbone CAs of C-alpha atoms of wild type and mutant variants. **c** Comparison distance between center of mass of ocriplasmin and substrate; It shows the effective vicinity of wild type rather than mutant variants of ocriplasmin. Wild type (blue) and mutant variants, proteolytic (red), autolytic (green) and mixed (dark violet) (R. Baghban, unpublished work)

The RMSF values of the active site located residues, i.e. His61, Asp104, and Ser199, in the mutant structure of ocriplasmin-substrate complex were greater than that of the wild type. These values were relatively stable in the wild protein structure compared to in the mutant types, mainly owing to more interactions between the substrate and wild-type ocriplasmin. The results are shown in Fig. 2a-c.

### Construction of Ocriplasmin Variants

**Fig. 3:**
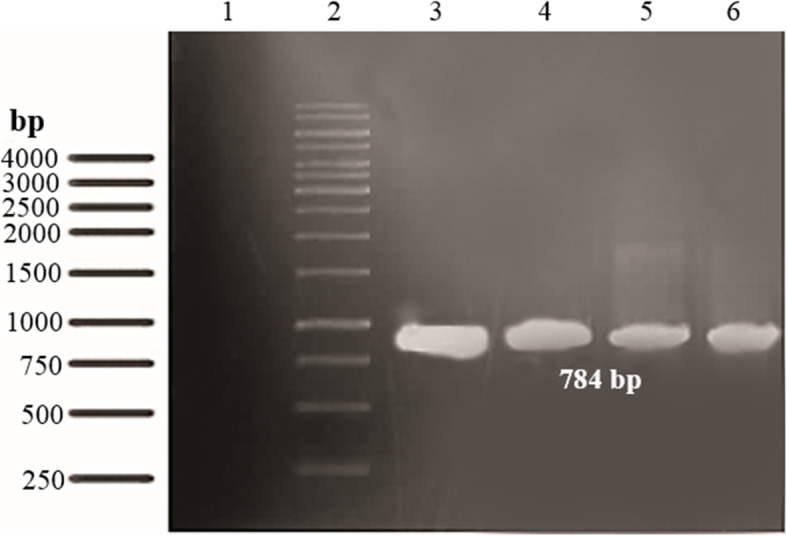
Confirmation of recombinant wild type and mutant clones screened by PCR. Column 1: Negative control, Column 2: DNA ladder, Column 3: PCR products (784 bp) of wild type, Column 4–6: PCR products (784 bp) of autolytic, proteolytic and mixed variants, respectively

Table 1 presents the sequences of primers used for this recombination. The synthesized ocriplasmin gene and the three mutant ocriplasmin variants were cloned in the pPinkα-Hc plasmid downstream from the AOX1 promoter after being generated through site-directed mutagenesis. Then the linearized pPinkα-Hc-ocriplasmin vectors were introduced into *P. pastoris* cells and positive clones were confirmed using PCR (Fig. 3).

**Table 1:** The sequences of the primers used for amplification of Ocriplasmin variants

Variants	5′ sequence 3′
Wild	α factor-F1: CTATTGCCAGATTGCTGC CAC1-R1: GCGTGAATGTAAGCGTGAC
Proteolytic	OCR-F2: GGTGCTGGTTTGTTGGAAGAAGCTCAATTGC OCR-R2: CAATTGAGCTTCTTCCAACAAACCAGCACCG
Autolytic	OCR-F3: AGAATGGGTTTTGACTACTGCTCACTGTTTGG OCR-R3: CCAAACAGTGAGCAGTAGTCAAAACCCATTCT
Mixed	OCR-F4: CGGTGCTGGTTTGTTGGAAGAAGCTCAATTGC OCR-R4: GCAATTGAGCTTCTTCCAACAAACCAGCACCG

### Expression, Optimization and, Characterization of Ocriplasmin Variants in *P. pastoris*

Recombinant plasmids with the wild-type and plasmids-containing mutated versions of the gene were transformed into *P. pastoris* cells. *PichiaPink™* is ADE2 auxotroph that is n^,^t able to grow in the absence of adenine because the full deletion of the ADE2 gene. The pPinkα-HC plasmid contains a functional ADE2 gene for complementation, requiring multiple genomic integration events to permit adequate ADE2 and resultant adenine production. Mutation in the ADE2 gene leads to the accumulation of precursors of purine in the vacuole leading to the formation of pink-colored colonies [33, 34]. The color intensity of colonies generally correlates with the levels of protein expression. The pink colonies are negative and express very little ADE2 gene product, but white colonies as positive clones express a greater transformed gene construct through stable integration of the recombinant vector into the host cell genome.

**Fig. 4:**
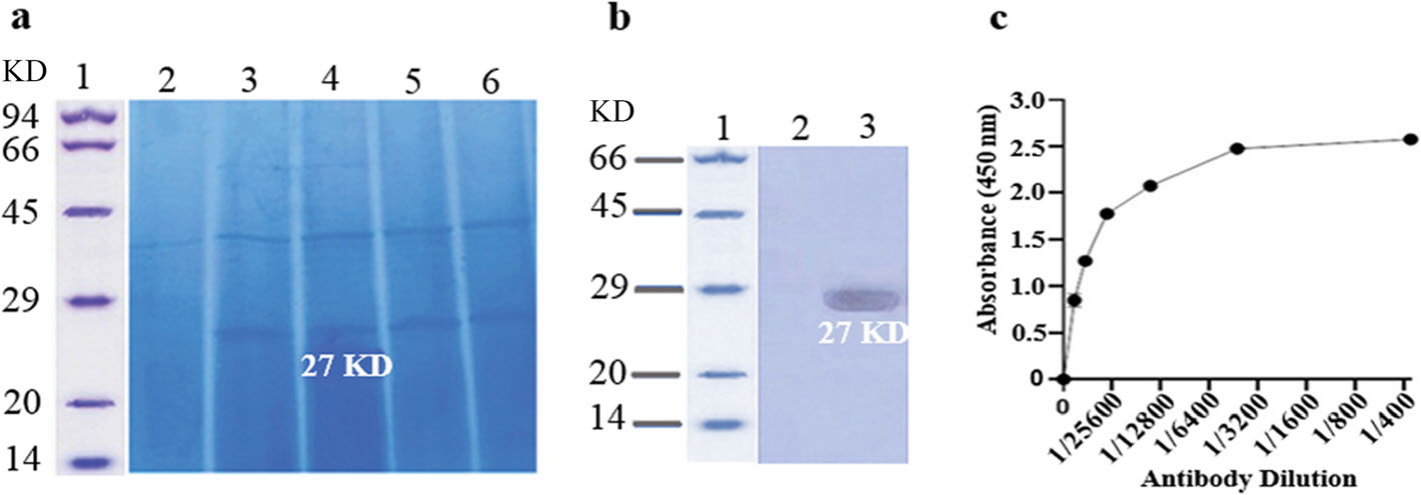
Expression and characterization of ocriplasmin variants in *P. pastoris*. **a** Expression of ocriplasmin after optimization in *P. pastoris.* Column 1: Protein marker, Column 2: Negative control, (*P. pastoris* none transformed) Column 3–6: a 27 KDa protein product relating wild-type, autolytic, proteolytic and mixed variants, respectively. **b** Western blotting, Column 1: Protein marker, Column 2: Negative control, Column 3: Purified ocriplasmin (a 27 KDa protein product) expressed in *P. pastoris*. **c** Elisa assay using anti-plasminogen antibody and recombinant protein expressed in *Pichia pastoris*

The secretory expression of all variants was performed in the BMMY medium. Then, the effect of different factors was evaluated to obtain optimal culture conditions for the ocriplasmin expression yield. The highest yield of ocriplasmin, i.e. approximately 0.2 mg/ml with a 93.6% purity, was achieved at pH = 6, a temperature of 30 °C, a methanol concentration of 0.5–1%, a duration of 72 h, and a medium comprising 1% yeast extract, 2% peptone and 1% tryptone. The secretory expression and optimization of all the variants were analyzed and a 27 kDa protein band detected through SDS-PAGE related to the molecular weight of ocriplasmin as per Fig. 4a.

Immunoblotting confirmed this finding, and the complete protein was recognized in a denatured form with a single band of approximately 27 kDa using the anti-plasminogen polyclonal antibody as per Fig. 4b.

According to Fig. 4c, the specificity of ocriplasmin toward the antibody was determined using ELISA, which was performed with the anti-plasminogen antibody raised in rabbit and confirmed the functionality of the *P. pastoris* expressed recombinant ocriplasmin.

### Activation of Ocriplasmin Variants and Autolytic/Proteolytic Activity Measurement

**Fig. 5:**
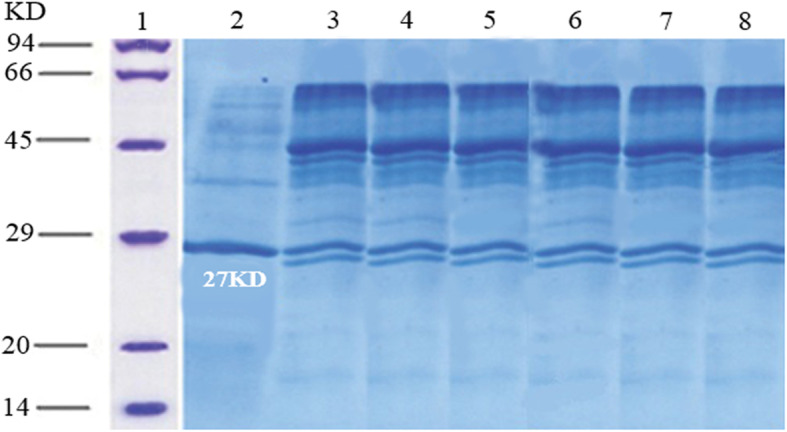
Activation of ocriplasmin by urokinase. Reducing SDS–PAGE illustrating that activation with urokinase is almost complete within 30 min. Ocriplasmin (27 KD) is converted into two fragments upon activation. Column 1: Protein marker, Column 2: Ocriplasmin before activation, Column 3–5: Samples collected 10, 30 and 60 min after addition of 2% urokinase. Column 6–8: Samples collected 10, 30 and 60 min after addition of 5% urokinase

Since the ocriplasmin and their variants expressed in the Pichia were in the inactive zymogen forms, it was necessary to be activated by the use of urokinase after purification. The activity of the aliquots collected at different times as well as the amount and rate of conversion into the active forms were monitored. This activity was maximized within 30 min in the empirical conditions applied and the conversion of zymogen and activity was increased simultaneously. The activation process was monitored by SDS–PAGE (Fig. 5).

### Hydrolytic Activity Assay

**Fig. 6:**
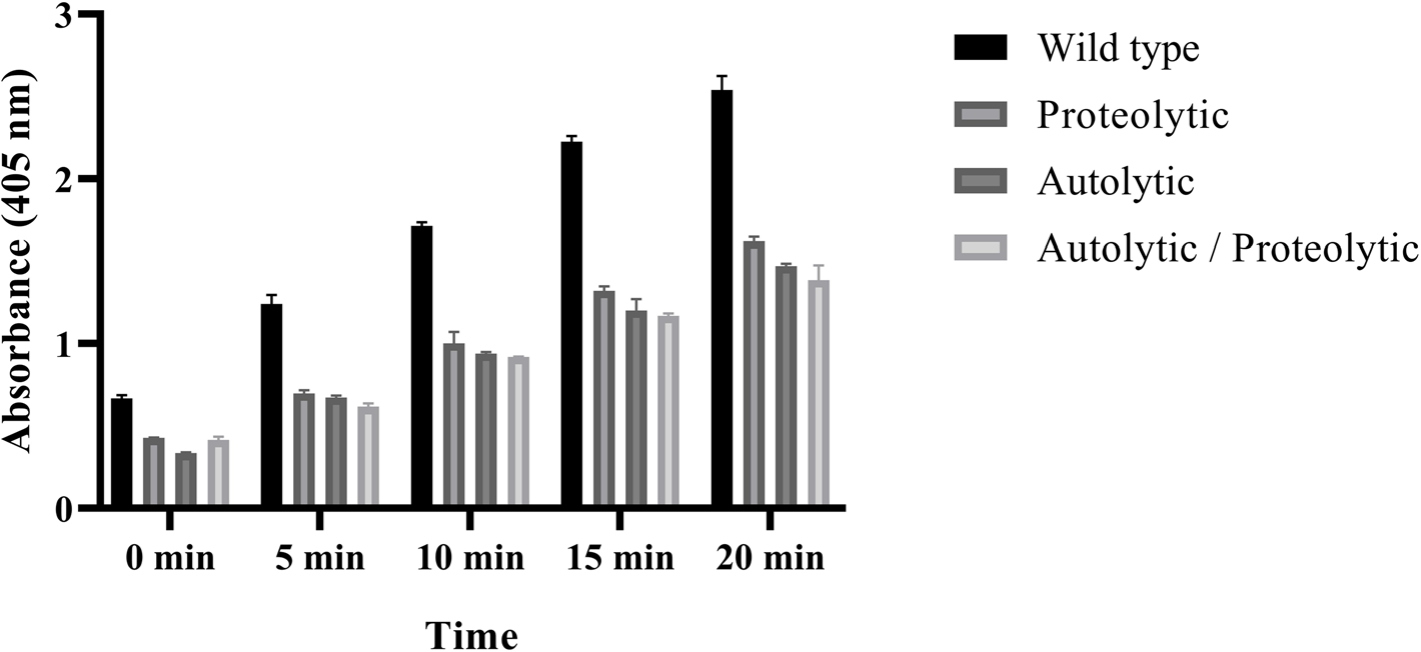
Hydrolytic activity assay of ocriplasmin variants measured at 405 nm. The product formation (color intensity) of mutant variants presented much rapid reduction, compared to the wild-type. Data indicate mean of duplicate measurements and analyzed by one-way ANOVA test for detecting significant differences compared to wild-type (*P* < 0.01). The bar graphs represent standard deviation of three independent assays that each well was assayed in triplicate

Fixed concentrations of enzyme variants were exposed to different concentrations of substrate for 0–20 min to form a colored (yellow) product. It has shown that the color intensity (product formation) in mutant variants has a much rapid reduction than wild-type. Reduced activity of mutant variants was also confirmed by reducing the optical density at 405 nm (Fig. 6). The results showed that A59T and K156E mutations have a significant effect on the hydrolytic activity of mutant variants compared to the wild type. These mutations reduced the enzyme catalyze ability.

### Kinetic Evaluations

**Fig. 7:**
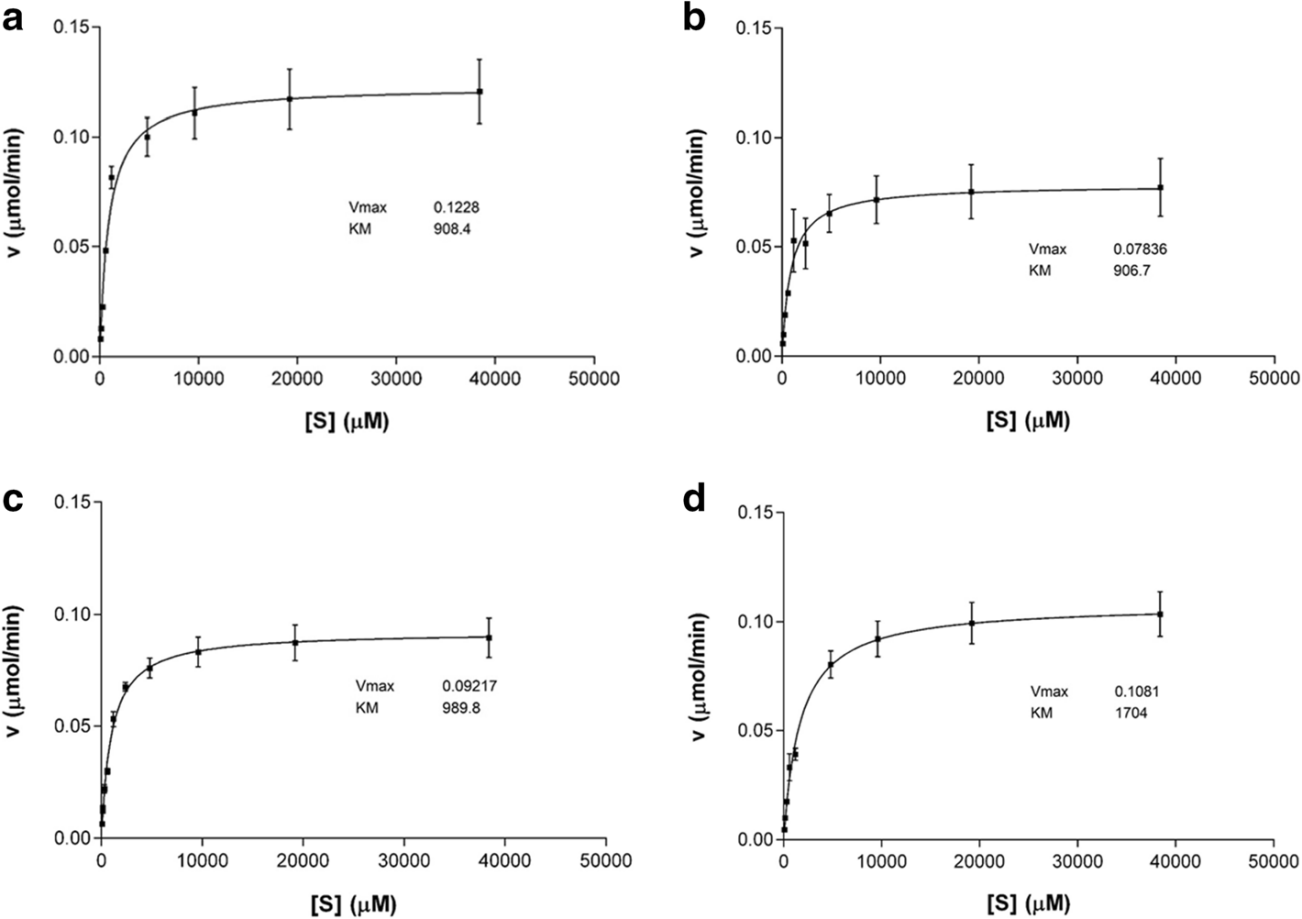
Kinetic parameters of the wild-type and mutant variants of Ocriplasmin. **a** wild-type, **b** proteolytic, **c** autolytic and **d** mixed variants with the substrate S-2403 on the basis of the Michaelis–Menten plot at 37 °C

According to Fig. 7a-d and the results presented in Table 2, the kinetic parameters of the wild-type and mutant variants of ocriplasmin with the substrate S-2403 were calculated based on the Michaelis–Menten plot at 37 °C. K_m_ was obtained as 908.4 μM for the wild variant of ocriplasmin, 906.7 μM for the proteolytic-type, 989.8 μM for the autolytic, and 1704 μM for the mixed variant. Given that the lowest K_m_ was associated with the proteolytic-type, this variant had a higher affinity to S-2403 compared to other variants. V_max_ of the wild-type (0.123 μmol/mg/min) was the highest compared to 0.0784, 0.092, and 0.1081 of the mutant variants. The hydrolytic efficiency of the wild-type ocriplasmin was, therefore, higher than that of the mutant variants.

**Table 2:** kinetic parameters of the wild-type and mutant variants of ocriplasmin with the substrate S-2403

Ocriplasmin variants Kinetic parameters	Wild	Proteolytic	Autolytic	Mixed
Vmax	0.123	0.0784	0.092	0.1081
Km	908.4	906.7	989.8	1704
Kcat	12.28	7.836	9.217	10.81
Kcat/Km	0.0135	0.0086	0.0093	0.0063

The values of the kinetic parameters obtained from the proposed model provided valuable knowledge on the enzyme nature. The rate of a reaction is halved compared to the maximum rate at a substrate concentration of K_m_. This constant also negatively relates to the substrate affinity to the enzyme. In other words, the higher the K_m_, the lower the affinity. As a result, the rate will be maximized at a lower substrate than at a lower K_m_. Besides, K_cat_ reflects the speed of a reaction, whereas K_cat_/K_m_ shows the efficiency of an enzyme in converting a substrate to a product. A nonlinear regression model can be employed to explain the relationship between the substrate concentration and the reaction speed based on Michaelis-Menten kinetics.

## Discussion

Ocriplasmin is a non-surgical alternative for the treatment of symptomatic vitreomacular traction. Its proteolytic activity at physiologic pH and natural vulnerability to autolytic inactivation can influence its efficacy in vivo and lead to serious damages [8].

In the present study, a series of site-directed mutagenesis performed in cDNA encoding ocriplasmin to reduce its autolytic and proteolytic activity, using two overlapping primers containing the desired mutations. Site-directed mutagenesis is a powerful tool to create specified pools of protein variants [35]. This technique has been effectively used in many studies to improve proteins and enzyme properties, such as; improved stability and specificity [36], generate mutants with increased thermostability, efficiency and half-lives compare to the wild-type enzyme [37], and improved activity towards substrate [13].

Among three autolytic cleavage sites in ocriplasmin (K156–E157, K166–V167, and R177–V178), lysine 156 was chosen to change to glutamic acid to reduce autolytic activity [27]. Previous studies revealed that initial cleavage occurs at position 156–157 because it is faster and after this cleavage, the sensitivity of the other two sites to autolytic cleavage greatly enhances [27]. Cleavage at 156–157 site was caused inactivation, and a mutant with cleavage at 177–178 site was detected in wild-types [8]. Therefore, K156E substitution is more effective in autolytic function rather than the other two.

Clinical data from Aoki et al. was used to design a variant to reduce the proteolytic activity of ocriplasmin. They investigated a patient with chronic thrombosis and the only abnormality was low plasminogen in plasma [38]. According to Miyata et al. the abnormality in plasminogen is because of Ala601Thr replacement in codon 601 of exon XV owing to the G to A nucleotide transition [39]. Val355Phe and Asp676Asn point mutations in the plasminogen gene also have been found in patients with dysPLGemia [40]. Given that ocriplasmin is the truncated version of human plasmin, and alanine 601 in plasmin is equal to alanine 59 in ocriplasmin, we hypothesized that substitution of alanine 59 with other amino acids (here, threonine) possibly will affect reducing the proteolytic activity of the enzyme.

Three ocriplasmin variants with A59T, K156E and A59T and K156E mutations simultaneously, were designed. Mutational analysis of ocriplasmin variants was performed by homology modeling, molecular dynamics simulations, and molecular docking studies. In silico mutational analysis of ocriplasmin revealed that A59T and K156E substitution have a significant effect on the reduction of proteolytic and autolytic activity, respectively. As expected, a reduced autolytic and proteolytic function was confirmed using docking study and MD simulation as a diminished binding affinity of mutant variants to the substrate rather than wild type.

After in silico analyses of designed mutant variants, wild-type and three mutant ocriplasmin (proteolytic, autolytic, and mixed variants) were constructed using site-directed mutagenesis and their expression in *P. pastoris* was studied and confirmed by western blot. A59T and K156E mutations reduced hydrolytic activity and the enzyme catalytic function. The product formation of mutant variants presented much rapid reduction, compared to the wild-type.

The kinetic parameters of wild type and mutant variants of ocriplasmin showed both variants with K156E substitution (autolytic and mixed variants) were more resistant to autolytic degradation than wild-type. These variants exhibited an increase in their Km values, leading to a decreased catalytic efficiency (the K_cat_/K_m_ ratio). The autolytic and mixed variants also exhibited reduced K_cat_ and V_max_ values. These results were in agreement with Noppen et al. [8]. Also, reduced K_cat_ and V_max_ values were observed in the variant with A59T mutation. The variants with both mutations showed the most increase in Km value (almost 2-fold) as well as reduced enzymatic affinity to the substrate. Thus, the results indicated that combined mutations at the ocriplasmin sequence were more effective compared with single mutations.

## Conclusions

Mutational analysis of ocriplasmin enzyme revealed that A59T and K156E mutagenesis could be used for the development of new ocriplasmin variants with higher stability and optimized activity. Further clinical evaluations could elucidate the potential higher therapeutic efficiency of these variants.

## Materials & Methods

### In Silico Methods for Mutational Analysis of Ocriplasmin Variants

The undesirable autolytic and proteolytic activities of ocriplasmin can be reduced with site-directed mutagenesis leading to an enzyme with improved catalytic activities and half-life. Three ocriplasmin variants were designed for improving biological/physicochemical characteristics. Using Swiss Model [41] and I-TASSER [42] web servers, 3D Structural Modelling of ocriplasmin was done. After that, the position of A59T and K156E mutations was studied by SPDBV [43] and PyMOL [44] software. Subsequently, the structural analyses of wild type and three mutants variants were performed using a protein interaction calculator (PIC) server available at crick.mbu.iisc.ernet.in/PIC [45]. A molecular docking simulation was performed using the freely available package Auto Dock 4.2.6 and Auto Dock Tools 1.5.6 software to study the interactions between substrate S-2403 (L-pyroglutamyl-L-phenylalanyl-Llysine-p-nitroaniline hydrochloride, Chromogenix, Milano, Italy, cat. 822,254–39 as a chromogenic substrate for plasmin, ocriplasmin, and streptokinase-activated plasminogen) and ocriplasmin variants and the binding capability was evaluated by calculating the free binding energy. The conformational features of protein-substrate complexes for all the variants were evaluated in a 100-ns Molecular Dynamics Simulation using the GROMACS 2016 package (R. Baghban, unpublished work).

### Construction and Cloning of the Ocriplasmin Expression Vector

The ocriplasmin coding sequence (DB08888 (DB05028)) was codon-optimized and synthesized (Genscript, U.S.A) for expression in *P. pastoris*. For subcloning into the expression vector, the ocriplasmin gene was amplified by polymerase chain reaction with the primers forward: 5′-CTATTGCCAGATTGCTGC-3′ and reverse: 5′-GCGTGAATGTAAGCGTGAC-3′ using Taq DNA polymerase (Vivantis, Gyonggi, South Korea). PCR protocol was carried out with initial denaturation at 94 °C for 4 min followed by, denaturation at 94 °C for 1 min, annealing at 55 °C for 45 Sec, extension at 72 °C for 45 Sec, and a final extension at 72 °C for 5 min. *Kpn*I and *Xho*I (TaKaRa Biotech, Japan) restriction enzymes were used to digest the purified PCR product and then ligated into the pPinkα-HC vector (Thermo Fisher Scientific, U.S.A) under the control of the alcohol oxidase I (AOX1) promoter. Afterward, the pPinkα-HC-ocriplasmin ligation product was transformed into the *E. coli* DH5-alpha (Thermo Fisher Scientific, U.S.A). To select the clones, the transformants were plated onto Luria-Bertani (LB) agar containing 100 mg/ml of ampicillin (Sigma-Aldrich, U.S.A). The recombinant constructs extracted from the ampicillin-resistant clones were confirmed with colony PCR and restriction digestion.

### Mutant Construction Using Site-Directed Mutagenesis

The optimized coding sequence of the ocriplasmin gene was employed as a template for the amplification. Glutamic acid was substituted for lysine 156 (K156E) in SOEing PCR to construct the mutant variant and reduce autolytic activities. Amplification was first performed on a 481 bp DNA fragment, containing the 5′ end of the ocriplasmin gene and upstream sequences (with α factor-F1 and OCR-R2 primers), and a 330 bp fragment, including the 3′ end of the gene and downstream sequences (with OCR-F2 and CAC1-R1 primers) following the PCR protocol of the previous section. To generate mutated fragments containing the desired point mutations, in the second round, amplified fragments were joined by overlap extension PCR method [46] according to the following temperature profile: initial denaturation at 94 °C for 5 min, annealing at 57 °C for 2 min, and extension at of 72 °C for 2 min. The reaction was followed by 30 cycles of denaturation at 94 °C for 1 min, annealing at 57 °C for 45 s, extension at 72 °C for 1 min, and a final extension at 72 °C for 5 min.

Alanine 59 changed to threonine (A59T) to construct the mutant fragment and reduce proteolytic activities. Amplification was performed on a 190 bp DNA fragment, including the 5′ end of the ocriplasmin gene and upstream sequences (with α factor-F1 and OCR-R3 primers) and a 617 bp fragment, including the 3′ end of the ocriplasmin gene and downstream sequences (with OCR-F3 and CAC1-R1 primers). Splicing by overlap extension was performed in the second round of PCR to join these fragments together [46] and generate a PCR fragment including the desired point mutations.

To produce the mutant fragment containing both the mutations, A59T and K156E, the fragment with A59T mutation was used as a template for adding K156E change as per the same steps described. These mutated PCR fragments were cloned into *Xho*I and *Kpn*I restriction sites of the pPinkα-HC vector and transferred into the *E. coli* DH5-alpha. PCR and plasmid digestion confirmed the cloning and sequencing confirmed the mutations.

### Transformation and Selection in *P. pastoris*

The recombinant pPinkα-HC-ocriplasmin vectors extracted and linearized with *Spe*I (TaKaRa Biotech, Japan). *P. pastoris* strain 4 competent cells (Thermo Fisher Scientific, U.S.A) were prepared and transformed with 5–10 μg linearized plasmids by electroporation for the wild type and three mutant variants of ocriplasmin. The transformations were recovered at 30 °C in yeast extract peptone dextrose with sorbitol (YPDS) for 2–12 h. The cell mixture spread on minimal dextrose (MD) plates were incubated at 30 °C for 3–10 days to obtain distinct colonies. Colony PCR was performed using specific primers to confirm the clones that integrated heterologous expression cassettes.

### Small Scale Expression and Optimization in *P. pastoris*

For the small-scale expression, the wild-type and mutant colonies inoculated from the fresh transformation plates in 10 ml BMGY medium (buffered glycerol-complex medium yeast) containing 1% (w/v) yeast extract, 2% (w/v) peptone, 100 mM potassium phosphate, pH 6.0, 1.34% (w/v) yeast nitrogen base (YNB), 0.0004% (w/v) biotin, and 1% (w/v) glycerol in a shaking incubator for 1–2 days at 24–30 °C until an optical density (OD600) of 4–6 was obtained. After transferring the cells into 50-ml of fresh BMGY medium, they were incubated for 1 day until obtaining an OD600 of 5–6. To induce the ocriplasmin expression, the cells were transferred to BMMY medium (buffered methanol-complex medium yeast with the same composition of BMGY except for glycerol, which is replaced with methanol). The induced cultures underwent a four-day incubation at 150 rpm and 30 °C and were fed, every 24 h, with methanol at a final concentration of 0.5%. The supernatant was collected at 24, 48, 72, and 96 h for analyzing the protein expression.

The culture conditions under which the ocriplasmin expression was optimized in the adenine-deficient *P. pastoris* strain included cell density = one and two-fold, pH = 3–7, temperature = 20, 25, and 30 °C, concentrations of glycerol = 5–10%, methanol = 0.25–8% and ammonium sulfate = 5–20% and induction time = 1–4 days. Sodium dodecyl sulfate-polyacrylamide gel electrophoresis (SDS-PAGE) and immunoblotting were performed to analyze the samples of the supernatants.

### Production of Anti-Plasminogen Polyclonal Antibody in Rabbit

Anti-plasmin antibodies can help detect ocriplasmin as a truncated version of recombinant human plasmin. A male New Zealand white rabbit was immunized with four intradermal injections on days 1, 14, 23, and 31. The first and the following three injections comprised a mixture of 150 μg of plasminogen (Merck, Germany) in 0.3 ml of saline respectively emulsified in 0.3 ml of Freund’s Complete Adjuvant and Freund’s Incomplete Adjuvant (Sigma-Aldrich, Germany). The blood samples collected from the ears of the rabbit before every immunization and kept for 2 h at 4 °C for coagulation. The serum separated from the coagulated blood was kept at − 20 °C. Western blotting and enzyme-linked immunosorbent assay (ELISA) were performed to quantitatively and qualitatively evaluate the produced antibody.

### Specificity Determination

The specificity of interactions between ocriplasmin expressed in *P. pastoris* and anti-plasminogen antibody produced in rabbits was determined using ELISA. The wells were coated overnight with 10 μg/ml of recombinant ocriplasmin in carbonate-bicarbonate buffer with pH 7.2 at 4 °C. The plates were rinsed three times using phosphate-buffered saline (PBS) with 0.05% Tween 20. 100 μl of different dilutions (1/400, 1/800, 1/1600, 1/3200, 1/6400, 1/12800, 1/25600) of rabbit serum containing the anti-plasminogen antibody was added to the wells. After one-hour incubation, the plates were rinsed and incubated for 1 h using 1: 5000 dilutions of the HRP-conjugated anti-rabbit antibody (Sigma-Aldrich, Germany). The TMB (Sigma-Aldrich, U.S.A) substrate was added after washing, and the reaction was stopped 20 min later using a 1 N H_2_SO_4_. The absorption was ultimately read at 450 nm.

### Immunoblotting

The recombinant ocriplasmin secreted from *P. pastoris* underwent immunoblotting by separating cell lysate proteins through SDS-PAGE and then transferring them to a polyvinylidene difluoride membrane (Thermo Fisher Scientific, U.S.A). A solution of 3% (w/v) bovine serum albumin in TBS (tris-buffered saline) with 0.1% Tween 20 blocked the membrane. Ocriplasmin was detected using an anti-plasminogen antibody produced in rabbit and HRP-conjugated goat anti-rabbit IgG as a secondary antibody (Sigma-Aldrich, U.S.A). Enhanced chemiluminescence (ECL) (Thermo Fisher Scientific, U.S.A) was then used to detect proteins.

### Activation of Ocriplasmin Variants

Since the ocriplasmin and their variants expressed in *Pichia* were in the inactive zymogen forms, it was necessary to be activated by the use of urokinase plasminogen activator (Sigma-Aldrich, U.S.A) to convert the purified ocriplasminogen variants into the corresponding active forms. We used different enzyme concentrations and incubation times to achieve the optimal time and concentration of urokinase enzyme for the activation of ocriplasmin. Solutions of the ocriplasminogen variants with concentrations of 5–20 mM were incubated at 37 °C for 10, 30 and, 60 min, in the presence of the urokinase at an ocriplasminogen to urokinase ratio of 100/2 and 100/5. After completing the phase of activation, SDS-PAGE was performed to evaluate the conversion of ocriplasminogen to the active form.

### Measuring Hydrolytic Activities

The hydrolytic activities of active ocriplasmin species extracted were monitored against S-2403™ (Chromogenix, Milano, Italy) as a substrate explained by Aerts et al. in 2012 [27]. The initial release rate of p-nitroaniline was monitored at 405 nm using Chromogenix S-2403™ to measure the hydrolytic activity of ocriplasmin. 1–10 nM ocriplasmin and 0.3 mM S-2403 substrate were used. Finally, the measured activities of wild type were compared with the hydrolytic activities determined for the mutant variants.

### Characterizing the Kinetic Parameters; the Michaelis Constant (K_m_), the Catalytic Rate (K_cat_), and V_max_

Initial hydrolysis rates were measured at different concentrations of S-2403 as the substrate to obtain the kinetic parameters of ocriplasmin variants. The mutant and wild-type enzymes were aliquoted in a duplicate manner to volumes of 75 μl to obtain a fixed concentration and the substrate was added at concentrations of 2.4, 1.2, 0.6, 0.3, 0.15, and 0.075 mM. After measuring the absorbance at 405 nm every 5 min, a standard curve was employed to calculate the micromoles of the released product per minute. Linear regression was used to calculate K_cat_, K_m,_ and V_max_ based on eq. (1), in which [S] represents the concentration of S-2403 and [OCR] that of active ocriplasmin. Moreover, K_cat_ and K_m_ could be calculated by analyzing total hydrolysis curves derived at [S] and V_max_ using eq. (2). The data were fitted to the Michaelis–Menten equation using GraphPad Prism software (version 8.0.2). The measurements were performed at 37 °C and in a mixture of 38 mM NaCl, 50 mM Tris-HCl, and 0.01% Tween 80 with pH = 7.4.

1 $$ {V}_i=\frac{k_{cat}.\left[\mathrm{OCR}\right].\left[S\right]}{K_m+\left[S\right]} $$

2 $$ {A}_t=\left[\left({A}_0-{A}_{\infty}\right).{e}^{-\frac{k_{cat}}{k_m}\left[\mathrm{OPL}\right]t}\right]+{A}_{\infty } $$

## Data Availability

All data and materials are within the paper.

## Abbreviations

VMA: vitreomacular adhesion
VMT: vitreomacular traction
PPV: pars plana vitrectomy
SDM: site-directed mutagenesis
PIC: protein interaction calculator
MDS: Molecular Dynamics Simulation
RMSD: root mean square deviation
RMSF: root mean square fluctuation
BMMY: Buffered Methanol-complex Medium
BMGY: Buffered Glycerol Complex Medium
MD plate: Minimal Dextrose plate
YPDS: yeast extract peptone dextrose with sorbitol
